# Prevalence and risk factors of fall-related injury among older adults in India: evidence from a cross-sectional observational study

**DOI:** 10.1186/s12889-022-12975-7

**Published:** 2022-03-19

**Authors:** Shobhit Srivastava, T. Muhammad

**Affiliations:** International Institute for Population Sciences, Mumbai, Maharashtra 400088 India

**Keywords:** Fall-related injury, Risk factors, Older adult, India

## Abstract

**Background:**

Falls and related injuries in older ages have become a major public health problem. This study aims to identify the prevalence of self-reported fall-related injury and to describe risk factors associated with fall-related injury among older adults in India.

**Method:**

The study used data from the "Building Knowledge Base on Population Ageing in India" (BKPAI), which was carried out in seven major states in India (2011). Bivariate and multivariable logistic regression analyses were conducted to examine the prevalence and risk factors of fall-related injury among older people.

**Results:**

The study found that 3.6% of older adults had a fall-related injury. Older adults with walk difficulty had a significantly higher likelihood of reporting fall-related injuries in comparison to their counterparts [adjusted odds ratio (AOR):1.80; confidence interval (CI): 1.38–2.36]. Older adults who consumed alcohol had significantly higher odds of reporting fall-related injuries than those who did not consume alcohol [AOR: 1.97; CI: 1.31–2.97]. Poor self-rated health was another risk factor for fall-related injury [AOR: 1.24; CI: 1.05–1.61]. Further, older adults with dementia were 2.15 times significantly more likely to report fall-related injuries than older adults with no dementia [AOR: 2.15; CI: 1.03–5.05]. Also, older women compared to men were 98% significantly more likely to report fall-related injury [AOR: 1.98; CI: 1.43–2.75]. The odds of reporting fall-related injury was significantly higher among those who had a secondary level education compared to those with no education [AOR: 1.44; CI: 1.01–2.06].

**Conclusions:**

Walking disabilities, alcohol consumption, poor self-rated health, dementia, and female gender were found to be the risk factors for fall-related injury among older adults. The results highlight the importance of improving physical as well as mental health of older individuals including dementia in terms of reducing the risk of experiencing fall-related injury.

## Background

With rapid aging of the global population, falls in older ages have become a major public health problem. Such falls among older adults are highly susceptible to injury due to high prevalence of diseases and age-related physiological changes later in life [1]. Fall-related injuries cause discomfort and disability for older adults as well as stress for caregivers [2]. Independent of other morbidity conditions, falls are associated with restricted mobility, decline in the ability to carry out day-to-day activities and an increased risk of admission in a nursing home [3].

A growing body of literature shows that falling limits an individual’s physical activity, social performance and increases the fear of falling and risk of repeated falling that ultimately leads to depression and reduction in quality of life [4–6]. Walking problems and having poor body balance were found as strong predictors of falls [7, 8]. Besides, inadequate use of materials, stumbling or slipping, and gait disturbances were also found as the common causes of falls in later ages [9]. Further, a review suggests that most falls occur due to multiple factors, including disorders of gait, balance, strength, and vision [10]. Since vision makes an important contribution to balance, impaired vision resulting from eye disease is a significant independent risk factor for falls and fractures in older people [11].

The use of excessive alcohol, on the other hand, is another risk factor for falls and related injuries. The study found that alcohol may interact with certain drugs to increase the risk of falls by producing changes in awareness, balance, and gait in older adults [12]. A longitudinal cohort study also revealed that consumption of 14 or more drinks per week is associated with an increased risk of subsequent falls in older adults [13]. Regardless of the amount of alcohol consumption, evidence shows that when alcohol is added to the equation of multiple medications and age-related disabilities, it can be dangerous to potentiate falls [14].

Cognitive impairment is another established risk factor for falls among older population [15]. Previous studies found that older adults with more severe forms of Alzheimer’s disease or dementia are presumably more likely to fall [9, 16]. Cognitive fluctuation, especially cognitive down periods, may also raise the incidence of falls and related injuries among older adults [17]. An analysis of the Study on global AGEing and adult health (SAGE) wave 1 data reveals that those who reported multiple chronic conditions and poor cognitive ability had a significantly higher likelihood of falling and report more fall-related injuries [18]. Those who reported falls in another study also had significantly higher chances of reporting poor self-rated health and more chronic conditions [19]. Although years lived by people have been increasing, they live with chronic conditions such as cardiovascular diseases, diabetes, and arthritis. These, along with the medication drugs used to treat them, can increase the fall risk among older individuals [20].

Although a few falls have a single cause, the majority result from interactions between long-term or short-term predisposing factors [21]. A study in Japan found that arthritis in the legs was significantly associated with falls among community-dwelling older men and women [22]. Similarly, long-term diseases such as diabetes and arthritis [23–25] and short-term factors such as stroke and other diseases and declines in functioning across short periods in later years of life [26–28] have been shown to increase the risk for fall-related injuries. Further, as evidence suggests, low socioeconomic status is associated with fall as it could lead to a poor household environment and untreated health conditions in older individuals [8, 29, 30]. A couple of community-based studies in India at different regional levels have also found female sex and increasing age to be major risk factors of falls among older population [31–33].

The prevalence of falls in Indian older adults in a review of 19 studies found to be ranging from 14 to 53% [34]. Although there is mounting evidence of the high burden of fall-related injuries in older people, there are only a few population-based studies that explored the prevalence and associated factors of fall-related injuries in India [35–37]. Thus, the present study aims to identify the prevalence of self-reported fall-related injury and to describe risk factors associated with fall-related injury among older adults using a large country-representative survey data in India.

## Data and methods

### Data

Data for this study were derived from “Building Knowledge Base on Population Ageing in India” (BKPAI), which was carried out in seven major states of India (Himachal Pradesh, Punjab, West Bengal, Odisha, Maharashtra, Kerala, and Tamil Nadu) that covered a total of 9852 older adults from 8329 households in rural and urban areas. The survey was conducted in 2011 [38]. These states had a higher percentage of population aged 60 years or older compared to the national average and also represent all regions of the country [38]. The individual questionnaire was used, which covers the socio-demographic profile, work history and benefits, income, and assets, living arrangement, social activities, the health status of older adults and social security-related question [38].

**Fig. 1:**
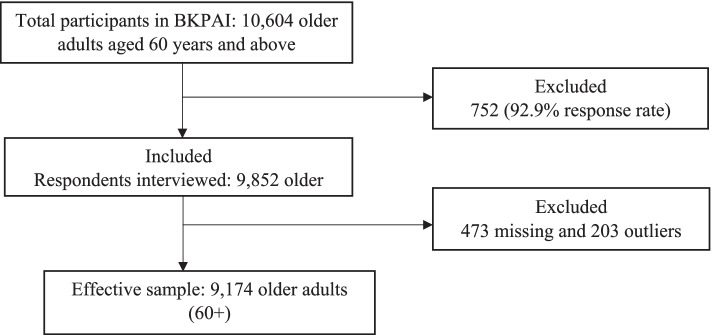
Sample selection for the present study

The BKPAI sample design entails a two-stage probability sampling where, first, villages were classified into different strata based on population size, and the number of Primary Sampling Units (PSUs) to be selected was determined in proportion to the population size of each stratum. Using probability proportional to population size (PPS) technique, the PSUs were chosen, and within each selected PSU, households were selected through systematic sampling [38]. Being the survey of older adults, the sample size was equally split between urban and rural areas, irrespective of the proportion of urban and rural population. Eighty Primary Sampling Units (PSU) (villages or urban wards) – 40 urban and an equal number of rural – with 16 households per PSU having an older person were covered in the survey [38] (UNFPA, 2012). The questionnaires for each state were bilingual, with questions in both the primary language of the states and in English. The survey was reviewed by the institutional review board, and informed consent was taken from the respondents before the survey. All the interviews were conducted in person. Out of total selected households, i.e., 8792 households, the response rate was 94.73%, and out of total selected individuals, i.e., 10,604 individuals, the response rate was 92.9%. In all, 9850 older adults who are aged 60 years and above were interviewed from 8329 households [38]. The final sample size for the analysis after removing missing cases (473 cases) and outliers (203 cases) was 9174 older adults (Fig. 1).

### Variable description

#### Outcome variable

The outcome variable, fall-related injury was measured by the question, “Has a doctor or nurse ever told you that you have a fall-related injury in the last year?” Thus, the reported fall-related injury among older adults in the current study was diagnosed by a doctor or nurse. The response was coded as “no” and “yes”.

#### Explanatory variable

Explanatory variables were selected according to the existing literature. Walking and vision disability was assessed through the questions “whether having difficulty in walking or vision?” The response was recoded as “no” and “partial/complete”. The walking and vision disability was diagnosed by a medical professional (doctor or nurse). Alcohol consumption was coded as “no” and “yes”. Self-rated health (SRH) was coded as “good”, which includes (excellent, very good, and good) and “poor”, which includes (fair and poor). Cognitive ability was measured using word recall and coded as high and low [39]. To measure cognitive ability, a scale of 0 to 10 was prepared, representing higher the score, better the cognitive ability [39]. Higher scores represent lower cognitive impairment and vice-versa. The words used for testing cognitive impairment were Bus, House, Chair, Banana, Sun, Bird, Cat, Saree, Rice, and Monkey [39]. Five or more words were recoded as 0, “high” representing better cognitive ability, and a score of four or less was recoded as 1 “low”, representing low cognitive ability [39, 40]. Further, Alzheimer’s and dementia were recoded as “no” and “yes”. Alzheimer’s and dementia were diagnosed by a medical professional (doctor or nurse). Diabetes and Arthritis were coded as “no” and “yes”. Both were diagnosed by medical professionals (doctor or nurse).

Furthermore, age was categorized as 60–69, 70–79, and 80 + years. Gender was categorized as men and women. Marital status was categorized as “not in union” included never married, widowed, divorced and separated, and “currently in a union”. Educational status was categorized as no education, below 5 years of schooling, 6–10 years of schooling, and 11 and above years of schooling [41]. Working status for the last year was categorized as no, yes and retired. Co-residence with children was categorized as “no” and “yes”.

Wealth status was computed using 30 household assets and was divided into five quintiles as poorest, poorer, middle, richer, richest [42]. Religion was categorized as Hindu, Muslim, Sikh, and others. Caste was categorized as Scheduled Caste, Scheduled Tribes, Other Backward Class, and others. Place of residence was categorized as rural and urban. Data for seven states were available in the data as mentioned in the data section.

### Statistical analysis

Descriptive statistics and bivariate analysis was used to find the initial results. A Chi-square test [43–45] was used to check the significance level between the prevalence of fall-related injury. Further, multivariable logistic regression analysis [46] was used to fulfil the objective of the study. All the covariates were adjusted in the model, and the model thus provides the adjusted estimates. The outcome variable ‘fall-related injury’ was recoded as 0 “no” and 1 “yes”. The results were presented in the form of adjusted odds ratio (AOR) with a 95% confidence interval (CI).

The model is usually put into a more compact form as follows:

$$\ln \left(\frac{P_i}{1-{P}_i}\right)={\beta}_0+{\beta}_1{x}_1+\dots +{\beta}_M{x}_{m-1},$$

Where, *β*_0_, …. . , *β*_*M*_ are regression coefficients indicating the relative effect of a particular explanatory variable on the outcome variable. These coefficients change as per the context in the analysis of the study.

The regression diagnostics for heteroscedasticity [47], multicollinearity [48], and outliers were performed via computation of variance inflation factors (VIFs) and visual inspection of residual plots for the regression models. The complex survey design effects were adjusted by using STATA *svyset* command. The whole statistical analyses were performed using STATA version 14 [49].

## Results

### Socio-demographic profile of older adults

The socio-demographic profile of older adults is presented in Table 1. It was found that 3.6% of older adults reported a fall-related injury. Nearly 23% of older adults had a partial or complete walking-related disability. About 59% of older adults had a partial or complete vision-related disability. Almost 8 % of older adults consumed alcohol. About 55% of older adults had poor SRH, and 60% of older adults had low cognitive ability. About 1 % of older adults had Alzheimer’s, and 1 % had dementia.

**Table 1:** Socio-demographic profile of older adults

Variables	Sample	Percentage
**Fall-related injury**		
No	8844	96.4
Yes	330	3.6
**Walking disability**		
No	7076	77.1
Partial/complete	2098	22.9
**Vision disability**		
No	3751	40.9
Partial/complete	5423	59.1
**Alcohol consumption**		
No	8474	92.4
Yes	700	7.6
**Self-rated health**		
Good	4094	44.6
Poor	5080	55.4
**Cognitive ability**		
High	3668	40.0
Low	5506	60.0
**Alzheimer’s**		
No	9059	98.8
Yes	115	1.3
**Dementia**		
No	9094	99.1
Yes	80	0.9
**Diabetes**		
No	8230	89.7
Yes	944	10.3
**Arthritis**		
No	6511	71.0
Yes	2663	29.0
**Age (years)**		
60–69	5665	61.8
70–79	2525	27.5
80+	984	10.7
**Gender**		
Men	4337	47.3
Women	4837	52.7
**Marital Status**		
Not in Union	3632	39.6
Currently in Union	5542	60.4
**Education**		
No education	4654	50.7
Below 5 years	1890	20.6
6–10 years	2070	22.6
11+ years	559	6.1
**Working status (last one year)**		
No	6172	67.3
Yes	2208	24.1
Retired	794	8.7
**Co-reside with children**		
No	2700	29.4
Yes	6474	70.6
**Wealth Status**		
Poorest	2168	23.6
Poorer	2024	22.1
Middle	1903	20.8
Richer	1708	18.6
Richest	1370	14.9
**Religion**		
Hindu	7297	79.5
Muslim	644	7.0
Sikh	847	9.2
Others	386	4.2
**Caste**		
Scheduled Caste	1896	20.7
Scheduled Tribe	515	5.6
Other Backward Class	3352	36.5
Others	3411	37.2
**Residence**		
Rural	6783	73.9
Urban	2391	26.1
**State**		
Kerala	1340	14.6
Himachal Pradesh	1456	15.9
Punjab	1240	13.5
West Bengal	1127	12.3
Orissa	1453	15.8
Maharashtra	1229	13.4
Tamil Nadu	1329	14.5
**Total**	9174	100.0

About one-tenth of older adults were from the age group 80 years and above. Almost 53% of older adults were women, and 47% were men. About 60% of older adults were currently in a union. Nearly 51% of older adults had no education, whereas 6 % had 11 and more years of education. Nearly 67% of older adults had not worked in the last year. About three in ten older adults were not residing with their children. About 24% of older adults belonged to households with poorest wealth quintile, and about 15% of older adults belonged to households with richest wealth status. Almost 80% of older adults were from the Hindu religion, and 21% were from the Scheduled Caste category. About 26% of older adults were from urban areas.

### Percentage of older adults suffering from fall-related injury by their background characteristics

Table 2 represents the percentage of older adults reporting fall-related injury by their background characteristics. It was found that the prevalence of fall-related injury was high among older adults with a partial or complete walking-related disability (6.1%) and partial or complete vision-related disability (4.2%). The prevalence of fall-related injury was high among older adults who consumed alcohol (5.7%). Older adults with poor SRH had a higher prevalence of fall-related injury (4.3%). Older adults with low cognitive ability (4.0%), with Alzheimer’s (7.3%), and with dementia (10%) had a higher prevalence of fall-related injury.

**Table 2:** Percentage of older adults suffering from fall-related injury by their background characteristics

Variables	%	***p*** < 0.05
**Walking disability**		*
No	2.9	
Partial/complete	6.1	
**Vision disability**		*
No	2.7	
Partial/complete	4.2	
**Alcohol consumption**		*
No	3.4	
Yes	5.7	
**Self-rated health**		*
Good	2.7	
Poor	4.3	
**Cognitive ability**		*
High	3.0	
Low	4.0	
**Alzheimer’s**		*
No	3.6	
Yes	7.3	
**Dementia**		*
No	3.5	
Yes	10.0	
**Diabetes**		*
No	3.5	
Yes	4.6	
**Arthritis**		*
No	3.3	
Yes	4.4	
**Age (years)**		
60–69	3.7	
70–79	3.4	
80+	3.9	
**Gender**		*
Men	2.8	
Women	4.3	
**Marital Status**		*
Not in Union	4.1	
Currently in Union	3.3	
**Education**		
No education	3.4	
Below 5 years	4.1	
6–10 years	3.8	
11+ years	2.9	
**Working status (last one year)**		*
No	3.8	
Yes	3.9	
Retired	0.9	
**Co-reside with children**		*
No	3.0	
Yes	3.9	
**Wealth Status**		*
Poorest	3.2	
Poorer	4.5	
Middle	4.7	
Richer	2.6	
Richest	2.8	
**Religion**		*
Hindu	3.4	
Muslim	5.6	
Sikh	2.1	
Others	7.5	
**Caste**		
Scheduled Caste	3.7	
Scheduled Tribe	2.7	
Other Backward Class	3.1	
Others	4.2	
**Residence**		
Rural	3.6	
Urban	3.5	
**State**		*
Kerala	5.7	
Himachal Pradesh	2.3	
Punjab	2.2	
West Bengal	5.3	
Orissa	1.7	
Maharashtra	7.8	
Tamil Nadu	1.0	

*if *p* < 0.05 based on chi-square test

Age did not show a significant association with fall-related injury among older adults. Older women had a higher prevalence of fall-related injuries (4.3%). Older adults who were not in a union had a higher prevalence of fall-related injury (4.1%). Older adults who worked in the last 1 year had a higher prevalence of fall-related injury (3.9%). Co-residence with children increased the risk of fall-related injury among older adults (3.9%). Older adults from the richest wealth quintile had the lowest prevalence of fall-related injury (2.8%). Older adults from Maharashtra (7.8%) and Kerala (5.7%) had the highest prevalence of fall-related injuries.

### Multivariable logistic regression estimates for fall-related injury among older adults by their background characteristics

Multivariable logistic regression estimates for fall-related injury among older adults by their background characteristics are presented in Table 3. Older adults with walk-related disability had an 80% significantly higher likelihood to suffer from fall-related injuries in comparison to their counterparts [AOR: 1.80; CI: 1.38–2.36]. Older adults who consumed alcohol had a 97% significantly higher likelihood to suffer from fall-related injuries in comparison to those who do not consume alcohol [AOR: 1.97; CI: 1.31–2.97]. Older adults who reported poor SRH had a 24% significantly higher likelihood to report fall-related injuries in comparison to those who reported good SRH [AOR: 1.24; CI: 1.05–1.61]. Older adults with Alzheimer’s had a higher likelihood to report fall-related injuries in comparison to older adults who did not have Alzheimer’s; however, the result was not significant. Dementia was a prime risk factor for fall-related injuries among older adults, i.e., older adults with dementia were 2.15 times significantly more likely to report fall-related injuries than older adults with no dementia [AOR: 2.15; CI: 1.03–5.05].

**Table 3:** Multivariable logistic regression estimates for fall-related injury among older adults by their background characteristic

Variables	AOR (95%CI)
**Walking disability**	
No	Ref.
Partial/complete	1.80*(1.38,2.36)
**Vision disability**	
No	Ref.
Partial/complete	1.06(0.81,1.38)
**Alcohol consumption**	
No	Ref.
Yes	1.97*(1.31,2.97)
**Self-rated health**	
Good	Ref.
Poor	1.24*(1.05,1.61)
**Cognitive ability**	
High	Ref.
Low	1.09(0.83,1.42)
**Alzheimer’s**	
No	Ref.
Yes	1.26(0.57,2.82)
**Dementia**	
No	Ref.
Yes	2.15*(1.03,5.05)
**Diabetes**	
No	Ref.
Yes	1.25(0.89,1.77)
**Arthritis**	
No	Ref.
Yes	1.15(0.88,1.49)
**Age (years)**	
60–69	Ref.
70–79	0.91(0.68,1.21)
80+	0.96(0.64,1.43)
**Gender**	
Men	Ref.
Women	1.98*(1.43,2.75)
**Marital Status**	
Not in Union	Ref.
Currently in Union	0.99(0.75,1.31)
**Education**	
No education	Ref.
Below 5 years	1.20(0.87,1.65)
6–10 years	1.44*(1.01,2.06)
11+ years	1.73(0.95,3.17)
**Working status (last one year)**	
No	Ref.
Yes	1.02(0.74,1.41)
Retired	0.88(0.51,1.53)
**Co-reside with children**	
No	Ref.
Yes	1.29(0.97,1.71)
**Wealth Status**	
Poorest	Ref.
Poorer	1.19(0.81,1.74)
Middle	1.28(0.85,1.92)
Richer	0.74(0.46,1.18)
Richest	0.60(0.36,1.02)
**Religion**	
Hindu	Ref.
Muslim	1.21(0.81,1.81)
Sikh	0.74(0.34,1.60)
Others	1.12(0.71,1.78)
**Caste**	
Scheduled Caste	Ref.
Scheduled Tribe	0.73(0.39,1.37)
Other Backward Class	0.78(0.54,1.14)
Others	0.94(0.68,1.3)
**Residence**	
Rural	Ref.
Urban	0.96(0.74,1.24)
**State**	
Kerala	Ref.
Himachal Pradesh	0.43*(0.26,0.71)
Punjab	0.52(0.27,1.01)
West Bengal	0.68(0.44,1.05)
Orissa	0.36*(0.21,0.60)
Maharashtra	1.71*(1.19,2.46)
Tamil Nadu	0.17*(0.08,0.34)

Ref: Reference; *if *p* < 0.05; *AOR* Adjusted Odds Ratio, *CI* Confidence Interval

Older women had 98% significantly higher likelihood of reporting fall-related injuries in comparison to older men [AOR: 1.98; CI: 1.43–2.75]. Older adults with 6–10 years of schooling had a 44% significantly higher likelihood for fall-related injuries in comparison to older adults with no education [AOR: 1.44; CI: 1.01–2.06]. Older adults with richest wealth status had a lower likelihood to report fall-related injuries, but the results were not significant. Older adults in Maharashtra had a 71% higher likelihood to report fall-related injuries in comparison to older adults from Kerala [AOR: 1.71; CI: 1.19–2.46].

## Discussion

Fall-related injury in later years of life is a major health concern, especially for those who have chronic conditions. Gait problems, a major contributor to the risk of falls and related injuries, can occur due to simple age-related changes in gait and balance as well as specific dysfunctions following a period of inactivity in old age [1]. Study shows that problems with mobility, balance, and loss of muscle strength contribute to the increased likelihood of falling [50]. Consistently, self-reported walking difficulty in the present study also emerged as an independent risk factor for fall-related injury among older adults. A couple of retrospective studies have also shown that imbalance and dizziness increased the risk of falling both among older men and women [51, 52]. Moreover, the bivariate analysis found that the older adults were prone to fall-related injuries if they had partial or complete vision difficulty. Similar findings have also been reported in studies that claimed that poor vision was a predictor of falls [53–55]. Even though, the multivariate analysis showed no statistical significance in the association of vision impairment and fall-related injuries in the present study.

Further, previous studies have shown that regular alcohol consumption may increase the fall risk in older adults through multiple mechanisms such as imbalance after acute alcohol ingestion, decreased cognitive function, and long-term impaired balance [56, 57]. In concordance with this, our finding also suggests that alcohol consumption increased the likelihood of fall-related injury among older people. Moreover, fall-related injury was significantly associated with poor self-rated health in the present study. In line with our finding, in a pooled analysis of two large Malaysian epidemiological studies, it was revealed that a lower rating of health was associated with an increased risk of falls [58]. Evidence also suggests that higher fall efficacy was more closely associated with better self-rated health [59].

Studies have shown severe cognitive impairment as an important risk factor for serious falls, and falls are associated with loss of independence in demented patients [15, 16, 60]. Nonetheless, as evidence suggests, the differentials in walking and balance might have partially accounted for the finding that older adults with Alzheimer’s disease/dementia were more likely to fall [17, 61]. Although we could not find a significant association of cognitive ability and Alzheimer’s disease with fall-related injury in our study population, dementia had shown a statistically significant association with fall-related injury in the multivariate regression model. Again, studies in India and other countries have shown that the fallers tended to have a greater prevalence of multiple chronic illnesses [22, 62, 63]. However, our study disclosed that there are no statistically significant differences in physical illnesses such as diabetes and arthritis between those who had fall-related injury and those who did not have.

Worldwide, in the older ages, sex difference was found in falling and women were more likely than men to be injured due to falls [30]. In a cohort-based study in South India, one in four older women fell compared with one in six men during the follow-up [31]. In line with the results of earlier studies, the present analysis also shows that there exists a statistically significant association of gender with a fall-related injury. Again, it is shown that a significant sex difference exists in the circumstances and injury potential when older adults fall indoors and outdoors [64]. The results of the current study also showed that people with 6–10 years education had 44% greater risk of fall-related injuries than those without any education. And those with 11+ years of education seem to have an even higher risk of fall-related injuries (although this is not significant but the 95% CI is relatively wider). The finding is at variance with multiple studies that show that the individuals with a lower educational level are particularly prone to falls and increased mortality due to fall injuries [34, 65, 66], indicating the need for further investigation.

Besides, factors such as religion, caste and household wealth status were not significant in the present study consistent with some previous studies [37, 67]. However, the low prevalence of fall-related injury among older adults who belonged to rich wealth quintiles observed in bivariate analyses shows the protective effect of higher socioeconomic status against falls and related injuries which is in parallel with past studies in India [8, 30, 34]. Huge regional variations in the prevalence of fall-related injury were also observed in our study. The states of Kerala, Maharashtra, and West Bengal were found to be the major states with a higher prevalence of fall-related injury among older population. This implicates that fall interventions have to be developed after identifying the different fall risks among older populations across states.

The study has certain limitations. Firstly, the cross-sectional design of the study does not necessarily demonstrate a causal relationship. Further, the reported fall-related injury might have recall bias where the participants, especially those cognitively impaired older adults, might have forgotten the fall-related injury or reported it more than once. Thus, under or over-reporting might have affected the current findings. Finally, the factors such as psychological problems and several chronic conditions are not considered in the present study. Although any strategy to reduce severe falls resulting in injuries requires assessing the critical behavioral determinants of an older individual’s risk for falling, due to unavailability of information, the physical activity, dietary patterns, and other behavioral factors also could not be analyzed in the present study. However, the strength of this study is its relatively large number of participants, which is representative at the national level. Also, the data with such comprehensive information is scarce in poor-resource settings like India, and the present study utilized such survey information for understanding the prevalence and risk factors of severe falls and related injuries among older Indian population.

## Conclusion

In this study, walking disabilities, alcohol consumption, poor self-rated health, dementia, and female gender were found to be the risk factors for fall-related injury among older adults. Hence, researchers and health practitioners should develop gender-based fall prevention strategies in older age especially among those who are at increased risk. The findings also highlight the importance of improving physical as well as mental health of older individuals including dementia in terms of reducing the risk of experiencing fall-related injury. Further retrospective and prospective studies are required for assessing several risk factors and their associations and planning appropriate prevention and control programs. Future studies are also warranted on cultural and behavioral factors that contribute to the differences in risk for falls and related injuries among several sub-populations in India.

## Data Availability

The study utilizes a secondary data which is available only on request from director@isec.ac.in or india.office@unfpa.org. The questionnaire and datasets generated and analysed during the current study are also available in the institute repository and accessible on request through http://www.isec.ac.in/.

## Abbreviations

BKPAI: Building Knowledge Base on Population Ageing in India
PSUs: Primary Sampling Units
PPS: Probability Proportional To Population Size
SRH: Self-Rated Health
VIF: Variance Inflation Factors
AOR: Adjusted Odds Ratio
CI: Confidence Interval
